# The sodium leak channel, NALCN, in health and disease

**DOI:** 10.3389/fncel.2014.00132

**Published:** 2014-05-20

**Authors:** Maud Cochet-Bissuel, Philippe Lory, Arnaud Monteil

**Affiliations:** ^1^Institut de Génomique Fonctionnelle, CNRS UMR 5203, Universités Montpellier 1&2Montpellier, France; ^2^INSERM, U 661Montpellier, France; ^3^LabEx 'Ion Channel Science and Therapeutics'Montpellier, France

**Keywords:** NALCN, UNC-79, UNC-80, ion channel, excitability

## Abstract

Ion channels are crucial components of cellular excitability and are involved in many neurological diseases. This review focuses on the sodium leak, G protein-coupled receptors (GPCRs)-activated NALCN channel that is predominantly expressed in neurons where it regulates the resting membrane potential and neuronal excitability. NALCN is part of a complex that includes not only GPCRs, but also UNC-79, UNC-80, NLF-1 and src family of Tyrosine kinases (SFKs). There is growing evidence that the NALCN channelosome critically regulates its ion conduction. Both in mammals and invertebrates, animal models revealed an involvement in many processes such as locomotor behaviors, sensitivity to volatile anesthetics, and respiratory rhythms. There is also evidence that alteration in this NALCN channelosome can cause a wide variety of diseases. Indeed, mutations in the *NALCN* gene were identified in *Infantile Neuroaxonal Dystrophy (INAD)* patients, as well as in patients with an *Autosomal Recessive Syndrome with severe hypotonia, speech impairment, and cognitive delay*. Deletions in *NALCN* gene were also reported in diseases such as 13q syndrome. In addition, genes encoding NALCN, NLF- 1, UNC-79, and UNC-80 proteins may be susceptibility loci for several diseases including bipolar disorder, schizophrenia, Alzheimer's disease, autism, epilepsy, alcoholism, cardiac diseases and cancer. Although the physiological role of the NALCN channelosome is poorly understood, its involvement in human diseases should foster interest for drug development in the near future. Toward this goal, we review here the current knowledge on the NALCN channelosome in physiology and diseases.

## Introduction

Ion channels are integral membrane proteins that allow specific ions to pass through lipid membranes following a concentration gradient (Hille, 2001). More than 400 genes are known that encode ion channel subunits. In addition, alternative splicing and heteromeric assembly of different subunits increase tremendously the variety of ion channels. They are involved in many signaling and control processes in the cell as well as in pathologies referred to as “channelopathies” (reviewed in Ashcroft, 2006; Camerino et al., 2008). In addition, pharmaceutical companies view ion channels as therapeutic targets of choice (Kaczorowski et al., 2008; Clare, 2010). In the present review, we focus on the *Na^+^-leak channel* (NALCN), a major player in determining the influence of extracellular Na^+^ on a neuron's basal excitability and its modulation by hormones and neurotransmitters.

### Structure of NALCN

**Figure 1:**
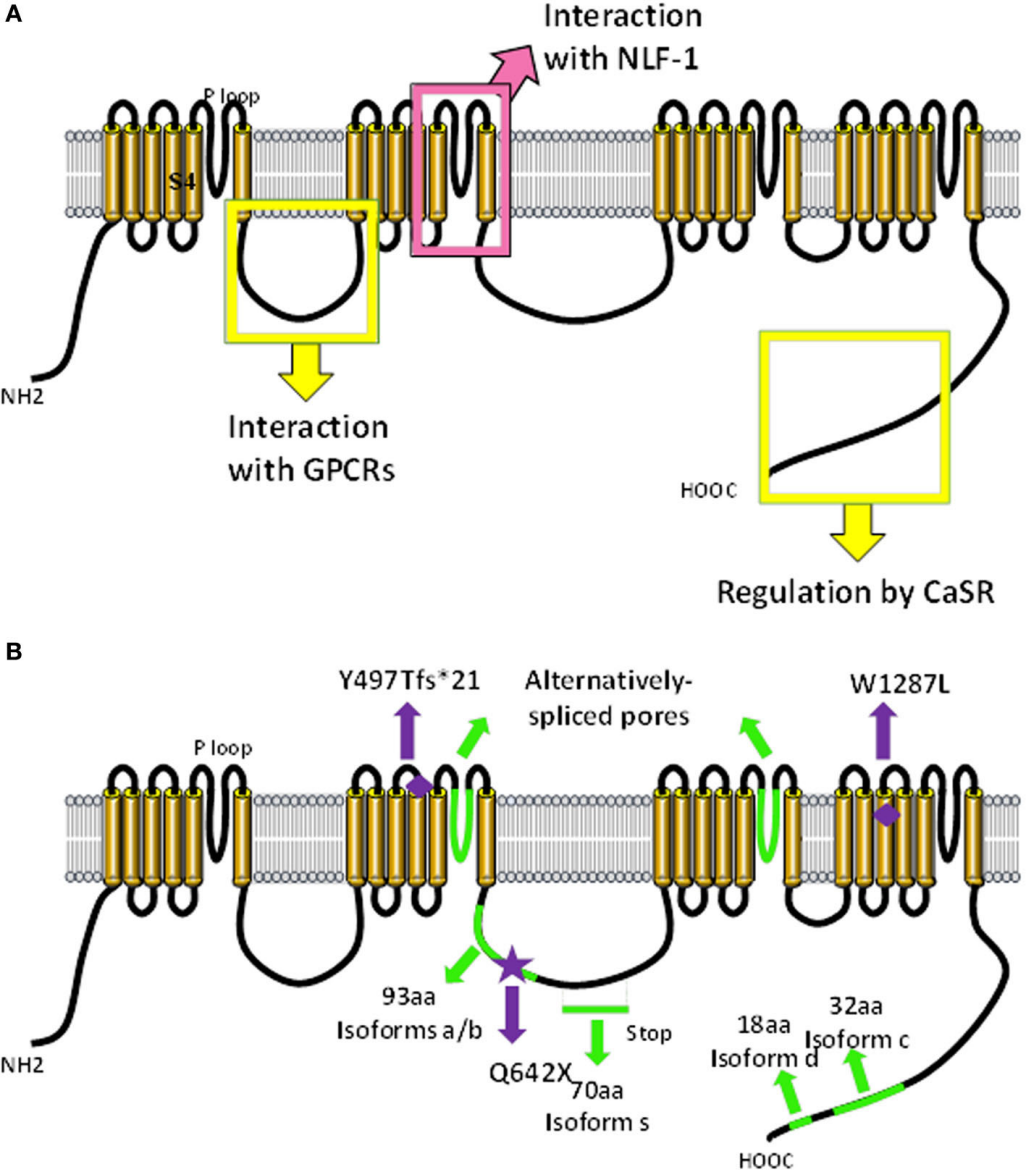
**Predicted molecular make-up of NALCN**. The predicted structure of NALCN is similar to α/α_1_ subunits of voltage-gated Na^+^ and Ca^2+^ channels (Lee et al., 1999). **(A)** It has four homologs repeats (domains I–IV) each composed of six transmembrane segments (S1–6). Four pore-forming loops (P-loops) spanning from S5 to S6 make up the ion selectivity filter. The intracellular loop linking domains I and II of NALCN interacts with M3R and molecular determinants involved in the regulation by CaSR are located in the carboxy-terminus (Swayne et al., 2009; Lu et al., 2010). The S5/P loop/S6 segment of transmembrane domain II is required for the interaction with NLF-1 (Xie et al., 2013). **(B)** Alternative events (*shown in green*) are detected in the intracellular loop linking domains II and III, in the P-loops of domains II and III, as well as in the carboxy-terminus (Senatore et al., 2013; *data not shown*). Mutations found in patients with INAD and an autosomal recessive syndrome with severe hypotonia, speech impairment, and cognitive delay are shown (Al-Sayed et al., 2013; Koroglu et al., 2013).

NALCN (also named Rb21, VGCNL-1, NA in *Drosophila melanogaster* and NCA-1/2 in *Caenorhabditis elegans*) was first cloned from rat brain and described by Perez-Reyes and colleagues who named it Rb21 (Lee et al., 1999). With the exception of the nematode *Caenorhabditis*, the cnidarian *Nematostella* and the sponge *Amphimedon* that have two related channels, there is only one gene encoding NALCN in other organisms (Liebeskind et al., 2012; Senatore et al., 2013). In mammals, NALCN is a 1738 amino-acids protein that forms the channel pore of the complex and has a predicted topology similar to voltage-gated sodium and calcium channels (Snutch and Monteil, 2007)(Figure 1A). Unlike other members of the four-domain ion channel family, the S4 transmembrane segments have fewer positive residues, especially in domains 2 and 4, possibly explaining NALCN's voltage insensitivity. The predicted pore region is also unique in that its ionic selectivity motif differs from that of calcium channels (EEEE or EEDD) and sodium channels (DEKA). NALCN's ion selectivity motif (EEKE) is implicated in its specific permeation properties. With the exception of *C. elegans* and *D. melanogaster*, alternative splicing events in the pore-forming region of invertebrate NALCN result in a calcium channel-like EEEE motif or a sodium channel-like EEKE (or EKEE) motif that remain to be explored at the functional level (Senatore et al., 2013). These findings suggest that NALCN could behave as a sodium or calcium channel depending on the expressed isoform (Senatore et al., 2013).

In humans, the gene encoding NALCN is located on chromosome 13q33.1 and comprises at least 44 exons (43 coding exons). Several splice variants were identified during our cloning step and by database scanning (*unpublished results* Figure 1B). Alternative splicing events were found in the intracellular loop linking domains 2 and 3 and in the carboxy-terminus region. Interestingly, there is a long non-coding RNA (lncRNA) gene named *NALCN*-AS1 partially overlapping with NALCN on the reverse strand (Gene ID: 100885778; http://www.ncbi.nlm.nih.gov/). lncRNAs are thought to function in various cellular contexts, including post-transcriptional regulation, post-translational regulation of protein activity, organization of protein complexes, cell–cell signaling, as well as recombination (reviewed in Geisler and Coller, 2013; Sabin et al., 2013). Importantly, lncRNAs would be associated with a wide range of neurodevelopmental, neurodegenerative and psychiatric diseases, as well as brain cancer (reviewed in Barry, 2014). The specific function of *NALCN*-AS1 remains to be elucidated.

## NALCN function

### Gating properties

The first functional characterization of NALCN was described by Ren and colleagues, who identified NALCN as the channel responsible for a tetrodotoxin (TTX)-resistant sodium leak current in mouse hippocampal neurons (Lu et al., 2007). In *Nalcn* knockout mice, they found that hippocampal neurons were hyperpolarized by ~10 mV compared to wild-type mice (Lu et al., 2007). They concluded that NALCN would contribute to the resting membrane potential in these neurons by eliciting a depolarizing current to counterbalance the hyperpolarizing current induced by two-pore potassium channels (reviewed in Ren, 2011; Lu and Feng, 2012). A similar NALCN-like channel activity was described in neurons from *L. stagnalis* and *C. elegans* (Lu and Feng, 2011; Xie et al., 2013). Contrasting with these data, NALCN does not conduct a background current but rather drives an acetylcholine-activated sodium current in the MIN-6 cell line, a pancreatic β-cell model (Swayne et al., 2009). This acetylcholine-activated NALCN current requires the M3 muscarinic receptor (M3R) and occurs though a G protein-independent, Src family of tyrosine kinases (SFK)-dependent pathway. Similarly, NALCN current was found to be activated by substance P (SP) and neurotensin through a SFK-dependent pathway in mouse hippocampal and ventral tegmental area neurons (Lu et al., 2009). It is not clear why NALCN behaves as a leak channel (e.g., looks like spontaneously active) in neuronal cells and not in MIN-6 cells and whether NALCN may be considered as a GPCR-activated channel with a cell type-dependent basal activity or as a constitutively open channel regulated by GPCRs. Although these studies provide the first demonstration for the functional properties and regulation of NALCN channels, much remains to be determined about the functionality of NALCN and the mechanism(s) responsible for its activation. Indeed, it has also been hypothesized that NALCN may not be an ion channel *per se* but rather an ion sensor (Senatore and Spafford, 2013). Discordance in the field indicates that more work is clearly required to reveal the real “channel” identity of NALCN. In the context of this review, we will consider NALCN as a pore-forming subunit.

### The NALCN channelosome

**Figure 2:**
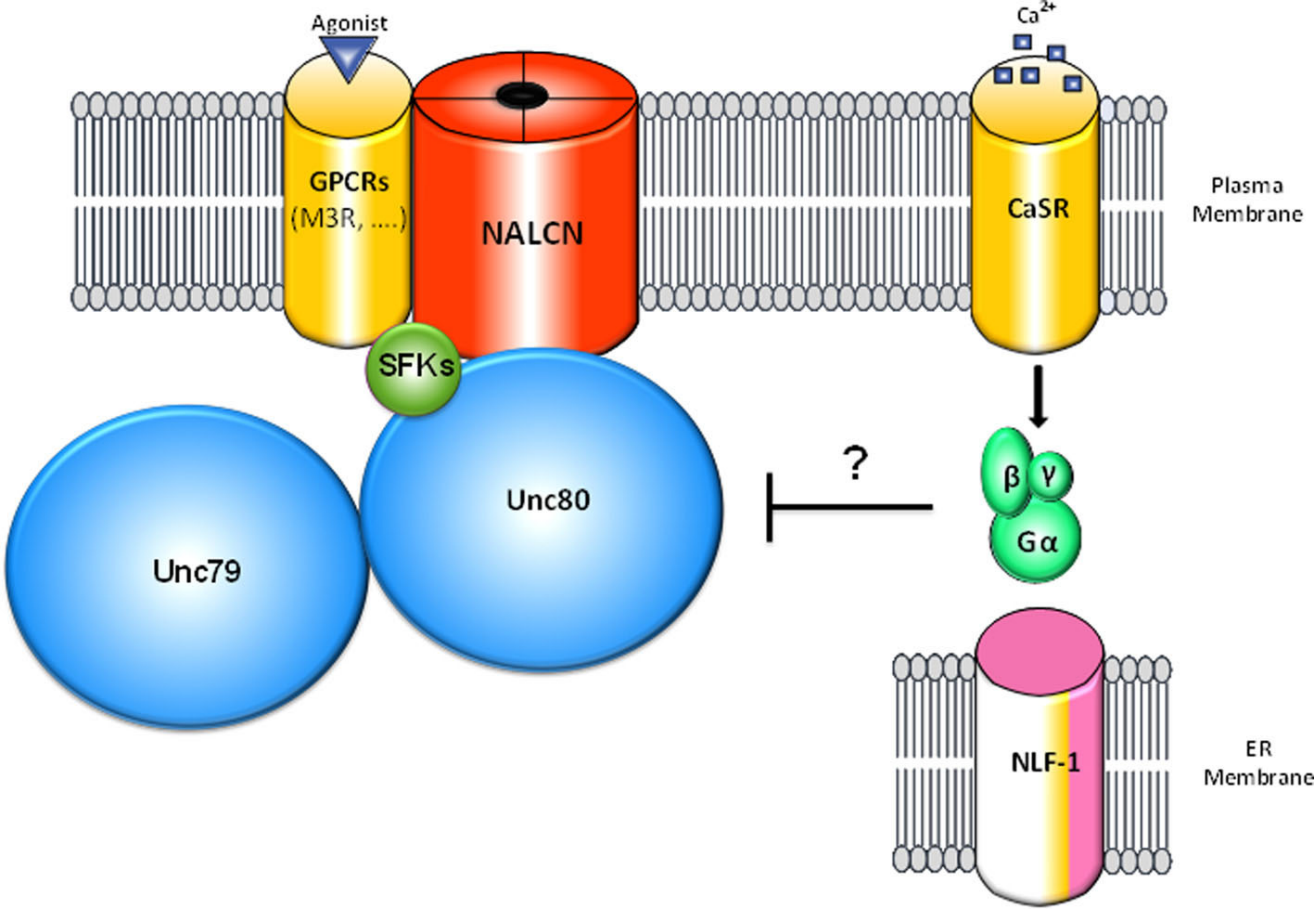
**Schematic representation of the NALCN channelosome**. The NALCN ion channel interacts with the M3 muscarinic receptor (M3R) that activates the channel through a G protein-independent and Src Family of Tyrosine Kinases (SFK)-dependent pathway upon activation by acetylcholine (Swayne et al., 2009). UNC-80 interacts with NALCN and SFKs and acts as a scaffolding protein (Wang and Ren, 2009; Lu et al., 2010). In addition, UNC-80 is involved in the expression levels of NALCN and UNC-79 as well as their neuronal localization (Jospin et al., 2007; Yeh et al., 2008). UNC-79 interacts with UNC-80 but not NALCN and is involved in the expression levels and the neuronal localization of NALCN and UNC-80 (Yeh et al., 2008). CaSR was found to regulate NALCN channel activity by an uncharacterized mechanism involving G-proteins activation and UNC-80 (Lu et al., 2010). For clarity, NLF-1, an endoplasmic reticulum-resident protein that interacts with NALCN and is possibly involved in its folding is shown apart. It is not known whether the interactions mentioned above are direct or not.

Like many ion channels, NALCN is associated with several proteins to form a larger channel complex (Figure 2, Table 1). These interacting proteins are involved in the folding, stabilization, cellular localization, and activation of NALCN.

**Table 1:** **Genes involved in the NALCN channelosome and their known roles**.

**Gene**	**Cytogenetic location**	**Coordinates (GRCh37 genome assembly)**	**Gene product function**	**References**
*NALCN*	13q33.1	101,706,129–102,068,812	Ion channel	Lu et al., 2007, 2009; Swayne et al., 2009
*UNC-80*	2q34	210,636,716–210,864,023	Scaffold protein for the SFKs and UNC-79	Wang and Ren, 2009; Lu et al., 2010
			Neuronal localization	Jospin et al., 2007; Yeh et al., 2008
*UNC-79*	14q32.12	93,799,565–94,174,222	Expression level of NALCN and UNC-80	Humphrey et al., 2007; Yeh et al., 2008; Lu et al., 2010
			Neuronal localization	Yeh et al., 2008
*NLF-1*	13q33.3	107,822,318–108,519,083	Expression level of NALCN	Xie et al., 2013
*CHRM3*	1q43	239,549,876–240,078,750	NALCN activator	Swayne et al., 2009
*SRC*	20q12-q13	35,973,088–36,033,835	Tyrosine kinase	Lu et al., 2009; Swayne et al., 2009

#### UNC-80

*UNC-80* (*also named* KIAA1843, c2orf21) is located on human chromosome 2q34 and has at least 45 exons. UNC-80 is a large protein of about 3300 amino acids without any predicted transmembrane segments or particular functional domains. UNC-80 contributes to the neuronal localization and/or stabilization of NALCN channel in *C. elegans* and *D. melanogaster* (Jospin et al., 2007; Yeh et al., 2008; Lear et al., 2013). In addition, UNC-80 acts as a scaffold protein for the SFKs and UNC-79. In fact, NALCN can interact with UNC-80 in the absence of UNC-79 and UNC-79 can interact with UNC-80 in the absence of NALCN. The interaction of NALCN and UNC-80 is required for the NALCN activation/inhibition by GPCRs in neurons (Wang and Ren, 2009; Lu et al., 2010). Similar to *NALCN*, databases indicate the existence of several *UNC-80* splice variants.

#### UNC-79

*UNC-79* (*also named* KIAA1409) is located on human chromosome 14q32.12 and has at least 48 exons. UNC-79 is also a large protein (~2800 amino acids) without any predicted transmembrane segments or particular functional domains. UNC-79 was found to be involved in regulating the neuronal localization of the NALCN channel complex both in *C. elegans* and *D. melanogaster* (Humphrey et al., 2007; Yeh et al., 2008; Lear et al., 2013). UNC-79 has been found to regulate the expression level of UNC-80 and, as a pair, UNC-79 and UNC-80 regulate the expression level of NALCN. This likely occurs through a post-transcriptional mechanism as no difference in the corresponding mRNA levels was found (Humphrey et al., 2007; Yeh et al., 2008). This regulation may not be conserved in mammals. As a matter of fact, *Unc-79* mutant mice lack detectable levels of the UNC-80 protein but NALCN is still present (Lu et al., 2010; Speca et al., 2010). Another difference between invertebrates and mammals concerns the redundant functions of these proteins. Indeed, in *D. melanogaster*, transgenic expression of NALCN, UNC-79, or UNC-80 does not relieve the requirement for the other subunits while, in mouse primary neurons, UNC-80 can bypass the requirement for UNC-79 (Lu et al., 2010; Lear et al., 2013).

#### NLF-1

NLF-1 (*NCA Localization Factor 1, also named* FAM155A) is an endoplasmic reticulum (ER) resident protein of around 438-468 amino-acids, depending on the species, that interacts with NALCN and promotes its neuronal localization in *C. elegans* (Xie et al., 2013). The gene encoding NLF-1 is located on human chromosome 13q33.3 and contains 3 exons. An intronic and non-coding transcript gene, *FAM155A*-IT1, is described on the same strand (Gene ID: 100874375; http://www.ncbi.nlm.nih.gov/) with no known function. In *C. elegans*, the loss of function of *nlf-1* results in a reduced leak current and a hyperpolarized resting membrane potential in premotor interneurons. NLF-1 function is conserved across species as the mouse homolog functionally substitutes to the *C. elegans* one. In addition, knockdown of the *D. melanogaster* ortholog gene, *CG33988* results in similar phenotypes as for *na* (see below; Ghezzi et al., 2014). Also, knockdown of *Nlf-1* in primary mouse cortical cultures effectively reduced the background Na^+^ current. It has been postulated that NLF-1 could function as a chaperone to facilitate the folding and assembly of NALCN channels. This interaction involves the second transmembrane domain of NALCN and requires its S5/P loop/S6 segment. The topology of NLF-1 is predicted to have one single transmembrane domain within 40 residues of the carboxy-terminus and lack known amino-terminal signal sequences. There are putative ER retention motifs (RXR) at the amino-terminus region. *nlf-1* is expressed in sensory neurons, interneurons, and excitatory motor neurons in *C. elegans*. Endogenous UNC-79 level is also significantly decreased in *nlf-1* mutants but the NLF-1 level is unaffected by the absence of UNC-79 and UNC-80. In humans, there is another gene homologous to *NLF-1, FAM155B* that is localized to Xq13.1 and encodes for a putative protein of 473 amino-acids. It is not known if this putative protein belongs to the NALCN channel complex. Both NLF-1 and FAM155B have a cysteine-rich domain (CRD) involved in signal transduction in other proteins (Pei and Grishin, 2012). In addition, these two proteins are related to Mid1, a fungal stretch-activated calcium channel component that forms a complex with the transmembrane calcium channel Cch1 (Maruoka et al., 2002).

#### G protein-coupled receptors (GPCRs)

In addition to its baseline activity, NALCN activity is enhanced/modulated by several GPCRs. Acetylcholine-induced NALCN current requires the interaction of NALCN and M3R proteins through the 1–2 loop of NALCN and the i3 and carboxy-terminus of M3R (Swayne et al., 2009). Other GPCRs, the Neurokinin 1 receptor (NK1R) and a neurotensin receptor that remains to be identified, activate NALCN upon the binding of their ligands SP and neurotensin in primary mouse pyramidal hippocampal neurons and dopaminergic neurons from the ventral tegmental area (Lu et al., 2009). However, whether NK1R interacts with NALCN remains to be shown. This raises the possibility that several GPCRs may activate NALCN and the challenge to determine the repertoire of NALCN-activating GPCRs persists. A full analysis of the literature gives some clues on this point. For example, it has been reported that 5-HT modulates a non-selective leak current involved in the resting membrane potential in the pre-Bötzinger complex and motor neurons likely through activation of the 5-HT_2A_ receptor (Ptak et al., 2009). Also, a cation conductance activated by glutamatergic metabotropic receptors through a G protein-independent pathway was described in CA3 hippocampal pyramidal neurons (Guerineau et al., 1995). In addition to its activation by several GPCRs, NALCN was found to be inhibited by another GPCR, the calcium-sensing receptor (CaSR) (Lu et al., 2010). This regulation is G-protein-dependent and SFK-independent, requires UNC-80 and involves molecular determinants in the carboxy-terminal portion of NALCN. It is not known if CaSR belongs to the NALCN channel complex.

## Expression pattern

*NALCN* is mainly expressed in the central nervous system (CNS) but also in heart, adrenal gland, thyroid gland, lymph node, and islets of Langerhans (both in α- and β-cells) (Lee et al., 1999; Kutlu et al., 2009; Swayne et al., 2009; Koroglu et al., 2013; Rorsman and Braun, 2013; see http://t1dbase.org). In the CNS, *NALCN* is expressed mainly in neurons, to a lesser extent in oligodendrocytes, and at a very low level in astrocytes (Cahoy et al., 2008). The temporal expression of *NALCN* is strongly correlated with the expression of genes involved in synapse development and with synaptic density in several areas of the human brain (Kang et al., 2011; see http://hbatlas.org/). The spatial expression of *NALCN* is also correlated with genes expressed in specific neural cell types such as *CALB1* (Cortical GABA interneurons), *CTGF* and *UNC-5C* (Subplate cortical glutamatergic neurons). In invertebrates, NALCN-orthologs are concentrated at the synapse in *D. melanogaster*, and along the axon in *C. elegans* and *L. stagnalis* (Nash et al., 2002; Humphrey et al., 2007; Yeh et al., 2008; Senatore et al., 2013). The cellular sub-localization of NALCN in mammals is not known.

## Physiological roles of NALCN: lessons from animal models

Mutations in genes coding for NALCN channel proteins, both in mammals and invertebrates, yield a wide range of phenotypes. Functional knock-out or hypomorphic mutations in these genes produce viable offspring (but fewer compared to wild-type) in *C. elegans* and *D. melanogaster*, whereas it is post-embryonic lethal in homozygous null mice (Krishnan and Nash, 1990; Nakayama et al., 2006; Lu et al., 2007; Speca et al., 2010). While no obvious developmental defects, including neuronal development, were found in *Nalcn* and *Unc-79* mutant mice, these mice die as a result of disrupted respiratory rhythm (Lu et al., 2007, see below). The depletion of *nca1/2, unc-79* or *unc-80* in *C. elegans* does not result in any gross abnormality in neuronal cell body position, neuronal processes, or fasciculation suggesting that these mutations do not interfere with the nervous system development (Pierce-Shimomura et al., 2008). We present below the phenotypes observed for the animal mutants in NALCN channel complex (Table 2).

**Table 2:** **Physiological roles of NALCN: lessons from animal models**.

**Phenotype**	**Gene**	**Species**	**Reference**
Locomotor activity	*nca1/2, unc-79, unc-80, nlf-1 na, unc-79*	*C. elegans D. melanogaster*	Sedensky and Meneely, 1987; Morgan et al., 1988; Krishnan and Nash, 1990Mir et al., 1997; Rajaram et al., 1999; Guan et al., 2000; Nash et al., 2002; Humphrey et al., 2007; Pierce-Shimomura et al., 2008; Yeh et al., 2008; Xie et al., 2013
Sensitivity to volatile anesthetics	*nca1/2, unc-79, unc-80 na, unc-79 unc-79*	*C. elegans D. melanogaster M. Musculus*	Morgan and Cascorbi, 1985; Sedensky and Meneely, 1987; Morgan et al., 1988, 1990 Krishnan and Nash, 1990; Nash et al., 1991; Campbell and Nash, 1994; Mir et al., 1997; Guan et al., 2000; Van Swinderen, 2006; Humphrey et al., 2007 Speca et al., 2010
Sensitivity to ethanol	*nca1/2, unc-79, unc-80 Unc-79*	*C. elegans M. musculus*	Morgan and Sedensky, 1995; Speca et al., 2010 Speca et al., 2010
Respiratory rhythm	*Nalcn nalcn*	*M. Musculus L. stagnalis*	Lu et al., 2007 Lu and Feng, 2011
Photic control of locomotion, circadian rythms	*na, nlf-1, unc-79, unc-80*	*D. melanogaster*	Campbell and Nash, 2001; Nash et al., 2002; Lear et al., 2005, 2013; Ghezzi et al., 2014
Abdominal morphology	*na, unc-79*	*D. melanogaster*	Krishnan and Nash, 1990; Mir et al., 1997; Nash et al., 2002; Humphrey et al., 2007
Social clustering	*na, nlf-1*	*D. melanogaster*	Burg et al., 2013; Ghezzi et al., 2014
Metabolism	*Unc-79*	*M. musculus*	Speca et al., 2010
Ethanol consumption	*Unc-79*	*M. musculus*	Speca et al., 2010
Systemic osmoregulation	*Nalcn*	*M. musculus*	Sinke et al., 2011
Pacemaker activity	*Nalcn*	*M. musculus*	Kim et al., 2012
Hyperactivity	*Unc-79*	*M. musculus*	Speca et al., 2010
Reproduction	*na*	*D. melanogaster*	Krishnan and Nash, 1990

### Locomotor activity

Wild-type *C. elegans* travels on a culture plate through the continuous and rhythmic propagation of sinusoidal body bends. A simultaneous loss of both *nca-1* and *nca-2*, or the individual loss of *unc-79, unc-80* or *nlf-1* results in fainting, a unique motor deficit characterized by periodic halting during movement (Sedensky and Meneely, 1987; Morgan et al., 1988; Rajaram et al., 1999; Humphrey et al., 2007; Jospin et al., 2007; Xie et al., 2013). A dysfunction of premotor interneurons was found to be the primary cause of the failure in the initiation and maintenance of rhythmic locomotion exhibited by fainters (Xie et al., 2013). A “hesitant” walking phenotype (e.g., the walk is interrupted by pauses) is also observed when *na* or *unc-79* are invalidated in *D. melanogaster* (Krishnan and Nash, 1990; Mir et al., 1997; Guan et al., 2000; Nash et al., 2002; Humphrey et al., 2007). Conversely, gain-of-function mutations in *nca-1* lead to exaggerated body bending, termed coiling, in *C. elegans* (Yeh et al., 2008).

When transitioned between solid and liquid environments, *C. elegans* switch between two patterns of rhythmic locomotion, crawling and swimming that are distinct in both kinematics and pattern of muscle activity (Pierce-Shimomura et al., 2008). A genetic screen was performed in order to find mutants capable of normal crawling but incapable of normal swimming. *unc-79, unc-80* and *nca-1;nca-2* mutants were found to be paralyzed upon immersion in liquid. The paralytic defect in swimming does not seem to be explained by general defects in neuronal excitability, synaptic function, or development because the fainting phenotype was not observed in mutants defective in voltage-gated calcium channels or major synaptic proteins.

### Sensitivity to general volatile anesthetics (GAs) and ethanol

Several studies reported an altered sensitivity to GAs both in invertebrates and mice mutants with some discrepancies. In *C. elegans, nca-1;nca-2, unc-79*, and *unc-80* mutants are hypersensitive to the immobilizing effect of halothane (~2–3 fold increase compared to controls) and other anesthetic agents (Sedensky and Meneely, 1987; Morgan et al., 1988, 1990; Humphrey et al., 2007). In fact, *unc-79* mutants are hypersensitive to thiomethoxuflurane, methoxyflurane, chloroform, and halothane. They are also more resistant to flurothyl and enflurane, insensitive to fluroxene and isoflurane, and mildly more sensitive to diethylether (Morgan and Cascorbi, 1985; Morgan et al., 1988, 1990; Humphrey et al., 2007). By contrast, hypomorphic *na^har38^* and *na^har85^* drosophila mutants were first described as resistant to halothane, methoxyflurane, chloroform and trichloroethylene but not to diethylether, isoflurane, and enflurane (Krishnan and Nash, 1990; Nash et al., 1991; Campbell and Nash, 1994; Mir et al., 1997). However, it was then reported that *unc-79* and *na* mutants are hypersensitive to halothane but not enflurane (Guan et al., 2000; Humphrey et al., 2007). The fact that *na* mutants were first described as resistant instead of hypersensitive may be explained by the method used to score anesthetic sensitivity but in some cases also by the fact that different alleles were tested. In another study, *na^har38^* mutation was found to be hypersensitive to isoflurane and to increase the potency with which isoflurane alters local field potentials recorded directly from fly brains (Van Swinderen, 2006). It was also shown that halothane presynaptically depresses synaptic transmission in wild-type drosophila larvae but not in *na^har38^* and *na^har85^* mutants (Nishikawa and Kidokoro, 1999). In mammals, heterozygous *Lightweight* mice, that carry a hypomorphic mutation in *Unc-79*, exhibit no alteration in Minimum Alveolar Concentration (MAC) in response to halothane, cyclopropane, or sevoflurane. However, a significant resistance to isoflurane-induced anesthesia was described (Speca et al., 2010).

It remains to be determined if the NALCN channel complex is a direct target for GAs, is important for the function of cells that contain such targets, or influences anesthesia more indirectly. GAs produce a widespread neurodepression in the CNS by enhancing inhibitory neurotransmission and reducing excitatory neurotransmission. However, the action mechanisms of GAs are not completely understood. Several ion channels and GPCRs are affected by these compounds (reviewed in Chau, 2010; Minami and Uezono, 2013). Of interest, it is known for a long time that GAs such as halothane, hyperpolarize neurons by acting on the potassium currents likely by activating two-pore potassium channels (Patel et al., 1999; Gruss et al., 2004; Liu et al., 2004). Considering that neurons from *Nalcn* knockout animals exhibit a ~10 mV hyperpolarization of their resting membrane potential, it is tempting to hypothesize that, in the case of an observed hypersensitivity, the absence of NALCN could remove a kind of brake for some GAs to act on their targets such as two-pore potassium channels. In this scheme, it would explain why, contrasting with *C. elegans* and *D. melanogaster unc-79* and *unc-80* mutants, heterozygous *lightweight* mice do not display any hypersensitivity to halothane. In fact, NALCN current and protein were found to be still present in *Unc-79* deficient mice whereas this is not the case in *C. elegans* and *D. melanogaster* (Yeh et al., 2008; Lu et al., 2010; Speca et al., 2010; Lear et al., 2013). An observed resistance to other GAs implies that the NALCN channel complex is a direct target while an absence of phenotype to other GAs would indicate that other targets beside NALCN are involved.

In addition to GAs, alteration in ethanol sensitivity was described in animal mutants for the NALCN channelosome. Morgan and Sedensky, in 1995, found mutations in several genes in *C. elegans* which seem to control the sensitivity to ethanol, including *unc-79* (Morgan and Sedensky, 1995). As a matter of fact, *unc-79* mutants showed a decrease by about 25% in ethanol sensitivity. By contrast, it was recently reported that *unc-79, unc-80*, and *nca-1;nca-2* mutants show hypersensitivity to ethanol (Speca et al., 2010). This hypersensitivity seems to be conserved in mammals as heterozygous *Lightweight* mice exhibit a highly significant increase in the sensitivity to the acute sedative effects of ethanol (Speca et al., 2010). Heterozygous *Lightweight* mice also present an increased ethanol preference and consumption, compared to wild-type mice, particularly at higher alcohol concentrations (Speca et al., 2010). As with GAs, the nature of the interaction between ethanol and NALCN physiology remains to be studied.

### Respiratory rhythm

In 2007, it was shown that the *Nalcn* gene is crucial for survival in mammals (Lu et al., 2007). Homozygous *Nalcn* knockout mice pups appear normal up to 12 h after birth and then die within 12 h due to severely disrupted respiratory rhythms (Lu et al., 2007). While wild-type mice had no abnormalities, knockout animals' respiration was highly sporadic. Breathing was characterized by ~5 s of apnea, followed by a burst of deep breathing for ~5 s, and this occurred at a rate of ~5 apnea events/minute. Interestingly, this pattern is reminiscent of the periodic breathing of Cheyne-Stokes respiration found in humans with CNS damage (reviewed in Strohl, 2003). Electrophysiological recording from the fourth cervical nerve root that innervates the diaphragm revealed that rhythmic electrical activity present in wild-type mice was largely absent. Thus, the defects observed in the respiratory rhythm in knockout mice are likely to reflect defects in electrical signaling in the nervous system (Lu et al., 2007).

More recently, Lu and Feng, 2011, investigated the properties of a *NALCN* ortholog in the snail *L. stagnalis* and its role in the activity of a respiratory pacemaker neuron (Lu and Feng, 2011). An *in vivo* investigation of its role on regulating respiratory behavior was performed by using RNA interference approaches. Animals in which *nalcn* was knocked down showed a reduced total breathing time compared to the naïve control. The resting membrane potential of the right pedal dorsal 1 (RPeD1) neuron that initiates the respiratory rhythm was found to be hyperpolarized by ~15 mV and its rhythmic firing was abolished. Thus, NALCN also plays a role in maintaining the respiratory activity in adult animal. It remains to be demonstrated if NALCN plays the same role in mammals by studying its functional properties in the preBötzinger complex and the retrotrapezoidnucleus/parafacial respiratory group that are involved in the respiratory rhythmogenesis (reviewed in Feldman et al., 2013).

### Photic control of locomotion, circadian rhythms

Several studies performed with *D. melanogaster* mutants revealed the NALCN channelosome as an important player of circadian rhythms. Indeed, null *na* mutants display disrupted circadian rhythm. Typically, wild-type *D. melanogaster* are diurnal, with a greater proportion of their activity occurring during the daytime but null *na* mutants exhibit most of their activity at night (Nash et al., 2002). Furthermore, light enhances the climbing deficit that is induced by GAs in these flies (Campbell and Nash, 2001). Interestingly, the amount of NA protein does not fluctuate over time or light cycles indicating that expression of NA is not under circadian regulation. The phenotype seems to be the result of a broad aberrant motor response to photic input. Central to this, *na* is expressed in circadian pacemaker neurons and regulates the output of these neurons (Lear et al., 2005). An accumulation of Pigment-Dispersing factor (PDF), a neuropeptide essential for robust circadian behavior, is observed in these neurons from *na* mutants. This suggests that *na* may be required for PDF release. It is not surprising to observe a disturbance of circadian rhythms in *na* null mutants as electrical activity is an important regulator of clock function both in invertebrates and mammals (Nitabach et al., 2002; Belle et al., 2009). Null mutations of *unc-79* and *unc-80* in *D. melanogaster* also display severe defects in circadian locomotor rhythmicity that are indistinguishable from *na* mutant phenotypes, and support the role of this channelosome in circadian rhythms (Lear et al., 2013). Knockdown of *nlf-1* in *D. melanogaster* was also found to phenocopy *na* knockdown for the circadian locomotion phenotype (Ghezzi et al., 2014).

### Social clustering

Involvement of the NALCN channelosome in social clustering, the natural tendency of animals of the same species to congregate in close proximity within a group, was recently described. Indeed, in a study aiming to investigate resource-independent local enhancement (RILE) in *D. melanogaster*, Burg et al., 2013, reported that *na^har38^* mutants display a deficient social phenotype compared to wild-type flies (Burg et al., 2013). This deficiency is rescued by the expression of *na* under the control of its native promoter. In addition, a complete rescue of the phenotype is also observed when *na* is expressed in cholinergic neurons and a partial rescue is obtained when *na* is expressed in glutamatergic neurons. *na* is strongly expressed in the mushroom body, a structure known to play a role in olfactory and visual learning and memory and the specific blockade of *na* expression in this structure affects RILE behavior. Knockdown of *nlf-1* in *D. melanogaster* was also found to significantly suppress the social clustering phenotype as for *na* (Ghezzi et al., 2014).

### Abdominal morphology (*narrow abdomen*)

The *na* mutant flies are noticeably smaller than controls and their abdomens are more slender and elongated but no obvious deformity has been identified (Krishnan and Nash, 1990; Mir et al., 1997; Nash et al., 2002). *D. melanogaster* bearing mutations in *unc-79* also exhibit a cylindric shaped abdomen (Humphrey et al., 2007). It is not known if this alteration represents a developmental defect or reflects altered physiology. However, *Nalcn* knockout mice neonates do not display gross abnormalities in embryonic development, righting responses, spontaneous limb movement, and toe/tail pinch responses (Lu et al., 2007).

### Metabolism (body composition and food consumption)

Heterozygous *Lightweight* mice are smaller (shorter in length and lower in body weight) and have a leaner body composition (increased lean tissue and decreased body fat) than wild-type mice (Speca et al., 2010). Interestingly, heterozygous *Lightweight* mice display an increased food intake in comparison with wild-type mice of the same weight.

### Systemic osmoregulation (serum sodium concentration)

A genetic analysis performed in mice demonstrated that *Nalcn* is involved in systemic osmoregulation by controlling the serum sodium concentration (Sinke et al., 2011). Furthermore, this study reported that heterozygous *Nalcn* knockout mice exhibit a significant hypernatremia.

### Pacemaker activity (interstitial cells of Cajal)

With others channels, such as transient receptor potential canonical (TRPC) channels, NALCN is partly responsible for the SP-induced depolarization and regulation of the intestinal pacemaking activity in the interstitial cells of Cajal (Kim et al., 2012). This activity generates the phasic contractions of the gastrointestinal muscles. Of note, in this study, the authors found that NALCN is not required for the basal pacemaking activity in ICCs.

## Possible implications of NALCN in human diseases

The NALCN channelosome was shown to be vital in mammals (Nakayama et al., 2006; Lu et al., 2007). Considering its role in regulating neuronal resting membrane potential, it is expected that polymorphisms, copy number variations (CNVs) and mutations in the corresponding genes may significantly impact neuronal physiology and lead to diseases. In this section, we review our current knowledge on data involving the NALCN channel complex in human diseases. We have also included a review of genetic data that loosely link the NALCN channel complex genes to diseases, which may provide insights into candidate genes involved in neuronal diseases (Table 3).

**Table 3:** **Possible implications of the NALCN channel complex in diseases**.

**Disease**	**Gene**	**Reference**
Infantile neuroaxonal dystrophy (INAD)	*NALCN*	Koroglu et al., 2013
Autosomal-recessive syndrome with severe hypotonia, speech impairment, and cognitive delay	*NALCN*	Al-Sayed et al., 2013
Cervical dystonia	*NALCN*	Mok et al., 2013
Cancer		
Pancreas	*NALCN*	Biankin et al., 2012
Non-small cell lung	*NALCN, UNC-80*	Lee et al., 2013
Tumor-derived endothelial cells	*NLF-1*	McGuire et al., 2012
Glioblastoma	*NALCN, NLF-1*	Fontanillo et al., 2012
Psychiatric disorders		
Bipolar disorder	*NALCN, UNC-79*	Baum et al., 2008; Askland et al., 2009; Wang et al., 2010
Schizophrenia	*NALCN*	Wang et al., 2010
Depression	*NLF-1*	Terracciano et al., 2010
Attention-deficit/hyperactivity disorder with conduct disorder	*NLF-1*	Anney et al., 2008
Epilepsy	*UNC-80*	Ratnapriya et al., 2010; EPICURE Consortium et al., 2012
Autism	*UNC-80*	Iossifov et al., 2012
13q syndrome	*NALCN, NLF-1*	Brown et al., 1995; Kirchhoff et al., 2009; Huang et al., 2012; Lalani et al., 2013
Alzheimer's disease	*UNC-80*	Scott et al., 2003 Lee et al., 2008; Grupe et al., 2007
	*UNC-79*	
Alcoholism	*NALCN*	Wetherill et al., 2014 Lind et al., 2010 Nurnberger et al., 2001; Schuckit et al., 2001
	*UNC-79*	
	*UNC-80*	
Restless legs syndrome	*NALCN*	Balaban et al., 2012
Primary biliary cirrhosis	*NLF-1*	Hirschfield et al., 2009
Hypertension	*NLF-1*	Adeyemo et al., 2009
Polyglutamine disorders	*NLF-1*	Whan et al., 2010

### Infantile neuroaxonal dystrophy (INAD)

Infantile neuroaxonal dystrophy (INAD) is a rare neurodegenerative disease characterized by progressive motor, mental and visual deterioration that begins in infancy. Onset is usually between the age of 6 months and 3 years and death typically ensues before the age of 10 years (reviewed in Gregory et al., 1993). Early clinical manifestations include bilateral pyramidal tract signs, truncal hypotonia, cognitive decline and optic atrophy. Most cases present with distal axonal swelling and spheroid bodies in tissue biopsies (Cowen and Olmstead, 1963; Morgan et al., 2006). Originally, this recessive disorder was linked solely to mutations in the *PLA2G6* gene, which encodes the calcium-independent phospholipase A2β (iPLA2β) enzyme, also designated iPLA2-VIA (Khateeb et al., 2006; Morgan et al., 2006). Recently, a study reported a mutation in the *NALCN* gene in two affected siblings with additional atypical features including facial dysmorphism, pectus carinatum, scoliosis, pes varus, zygodactyly and bilateral cryptorchidism. The patients also exhibit cerebellar atrophy and seizures and are still alive at 18 years-old (Koroglu et al., 2013; also see Seven et al., 2002). The identified mutation was a C to T conversion in *NALCN* coding exon 16 (c.1924C > T), creating a premature translational termination signal at codon 642 (Q642X) and truncated NALCN channel (Figure 1B). Interestingly, our unpublished data indicate that this exon is alternatively spliced resulting in the loss of one isoform but not NALCN in its entirety and may explain why the mutation is not lethal. It remains to be established whether this mutation results in a mRNA decay or expression of a truncated two-domain isoform. The fact that *PL2G6* and *NALCN* genes mutations result in a similar disease raises the possibility that iPLA2β could be a regulator of NALCN.

### Autosomal recessive syndrome with severe hypotonia, speech impairment, and cognitive delay

Mutations in the *NALCN* gene were recently reported in six patients with an autosomal-recessive syndrome characterized by severe hypotonia, speech impairment, and cognitive delay from two large consanguineous families (Al-Sayed et al., 2013). Three male patients (ranging from 4 to 7 years-old) from one family are homozygous for a single nucleotide deletion c.1489delT located in the coding region for the S4 helix from domain II (Figure 1B). This mutation results in a frame shift creating a stop codon 21 amino-acids downstream, which likely results in the production of a truncated protein or mRNA decay. Unless the involved exon is alternatively spliced, it is not clear why this deletion is not lethal as reported for *Nalcn* knockout mice (Lu et al., 2007). These patients have an absence of speech development, severe hypotonia, chronic constipation, global developmental delay, and facial dysmorphism which is reminiscent of infantile hypotonia with psychomotor retardation and characteristic facies (IHPRF, OMIM #615419). Three female patients (ranging from 9 to 17 years-old) from a second family are homozygous for a missense mutation c.3860G > T (W1287L) in exon 34 that encodes for the S4 helix from domain IV (Figure 1B). These patients have a milder but similar phenotype as the first family with additional features such as seizure disorder and hyperactivity.

### Cervical dystonia

Dystonia is a “syndrome of sustained muscle contractions, frequently causing twisting and repetitive movements or abnormal postures” (reviewed in Fahn et al., 1998). Estimates of the prevalence of dystonia range from ~1:10,000 to more than 1:200 (Nutt et al., 1988; Nakashima et al., 1995; Epidemiological Study of Dystonia in Europe Collaborative, G., 2000; Muller et al., 2002). Clinical presentation of dystonia is heterogeneous, from focal involvement such as cervical dystonia to generalized torsional dystonia. Cervical dystonia is the most common form of dystonia (Butler et al., 2004; Jankovic et al., 2007; Groen et al., 2013). The majority of cervical dystonia is transmitted in a non-Mendelian pattern, which suggests cervical dystonia is likely a complex disease rather than monogenic form. A recent genome-wide association study (GWAS) in 212 patients with this form of dystonia revealed a possible association with *NALCN* (Mok et al., 2013). However, this study did not find any single nucleotide polymorphisms (SNPs) with genome-wide significance (defined as *P* < 5 × 10^−8^) but a few clusters of possible association (defined as *P* < 5 × 10^−6^). Six SNPs within or in the vicinity of the *NALCN* gene with *P*-values ranging from 2.54 × 10^−6^ to 9.76 × 10^−7^ were found. Additional GWAS with larger cohorts of patients are required since the small sample size for this initial study was underpowered to detect loci with small disease effects.

### Psychiatric disorders

#### Schizophrenia and bipolar disorder

Schizophrenia (SCZ) is a chronic, severe disabling brain disorder characterized by abnormalities in the perception of reality. It most commonly manifests as auditory hallucinations, delusions, disorganized speech and thinking with significant social or occupational dysfunction (reviewed in Silveira et al., 2012). Bipolar disorder (BD), also known as manic-depressive illness, is a serious medical illness that causes shifts in a person's mood, energy, and ability to function (reviewed in Smith et al., 2012). Different from the normal ups and downs that everyone goes through, the symptoms of BD are severe. SCZ and BD affect around 1% of the population each. Both diseases have strong inherited components and growing evidence indicates that BD and SCZ may be closely related.

Interestingly, the *NALCN* gene lies within a region on chromosome 13q that has shown linkage to both BD and SCZ (reviewed in Detera-Wadleigh and McMahon, 2006). More specifically, the correlation between *NALCN* mRNA expression and the SCZ-associated gene *GABRB2* in human brain (Kang et al., 2011), along with the association between the *Drosophila* homolog, *na* with circadian rhythms (disruptions of which are a hallmark of BD) suggest that *NALCN* may play a role in these two disorders (reviewed in Lenox et al., 2002). Importantly, while some genetic studies point out *NALCN* as a susceptibility locus in SCZ and BD, other studies have not concurred with these findings. A GWAS performed by genotyping more than 550,000 SNPs in two independent cohorts of European origin (for a total of 1233 patients and 1439 controls) revealed a significant association between *NALCN* and BD (SNP rs9513877; Baum et al., 2008). This finding was confirmed by Askland et al. (2009). A genome-wide meta-analysis also identified a significant association of *NALCN* (SNP rs2044117) with both SCZ and BD in cohorts of 1172 (SCZ) and 653 (BD) European-American patients (Wang et al., 2010). However, no significant association between BD and *NALCN* was found in a Finnish cohort of 723 individuals from 180 families with type I BD (Ollila et al., 2009). Furthermore, there was no association of *NALCN* with SCZ in a cohort of 583 patients (Souza et al., 2011), nor in a group of 240 individuals with treatment-resistant SCZ (Teo et al., 2012). The discrepancies in candidate gene studies may partly result from the clinical and genetic heterogeneity of BD and SCZ and their complex inheritance model, but also from methodological issues such as the definition of clinical phenotype. More work is needed to confirm that *NALCN* is a susceptibility locus in BD and SCZ.

Several findings suggest that *UNC-79* and *UNC-80* are also associated with these disorders, further implicating the NALCN channelosome with SCZ and BD. The *UNC-79* encoding gene lies within a region on chromosome 14q that has shown linkage to BD. Askland et al., 2009, also suggested an association between SNP rs17125698 located at around 4Mb from the *UNC-79* gene and BD (Askland et al., 2009). Another study also reported an association of SNP rs11622475 located in 14q32 in BD (Wellcome Trust Case Control, C, 2007). However, this SNP is located at around 100Mb from *UNC-79*. A proteomic study suggested that UNC-80 could belong to the same protein complex as dysbindin, a SCZ susceptibility gene product (Mead et al., 2010).

#### Major depressive and attention-deficit/hyperactivity disorders

*NLF-1* has been loosely associated with both major depressive disorder and attention-deficit/hyperactivity disorder. Major depressive disorder (MDD) is a syndrome characterized by a number of behavioral, cognitive and emotional features. It is most commonly associated with a sad or depressed mood, a reduced capacity to feel pleasure, hopelessness, loss of energy, altered sleep patterns, weight fluctuations, difficulty in concentrating and suicidal ideation (reviewed in Uher et al., 2013). A GWAS of depression traits found a possible association between MDD and SNPs in the vicinity of *NLF-1* (SNPs rs9634463, rs7329003, rs713548, rs9301191, and rs1924397), but statistical significance was not reached for any gene (Terracciano et al., 2010). Attention-deficit/hyperactivity disorder (ADHD) is characterized by inattention, excessive motor activity, impulsivity and distractibility and affects 8–12% of school-age children worldwide (reviewed in Sharma and Couture, 2014). Individuals with ADHD show high co-morbidity with a wide range of psychiatric disorders. In a study aiming to identify susceptibility loci for ADHD with conduct disorder, Anney et al., 2008, did not find any significant association with any SNP but two of the strongest associations were found with SNPs rs10492664 and rs8002852 that are located in the vicinity of *NLF-1* (Anney et al., 2008). Interestingly, phenotypes observed for *Nk1R* knockout mice strongly parallel abnormalities expressed by patients with ADHD and heterozygous *Lightweight* mice are modestly but significantly hyperactive (Speca et al., 2010; Yan et al., 2010).

### Epilepsy

Epilepsy, a very common neurological disorder, is defined by the occurrence of unprovoked seizures caused by the synchronous discharge of large number of neurons (reviewed in Sander, 2003; Khan and Al Baradie, 2012). Abnormal expression or function of ion channels may be involved in the pathophysiology of both acquired and inherited epilepsy (reviewed in Mantegazza et al., 2010; Lerche et al., 2013). Considering the importance of the NALCN channel complex in setting the resting membrane potential of neurons, the NALCN channelosome gene mutations are clear candidates for epilepsy. As mentioned in this review, patients with INAD or an autosomal-recessive syndrome with severe hypotonia, speech impairment, and cognitive delay have seizures (Al-Sayed et al., 2013; Koroglu et al., 2013). Seizures were also reported for patients with chromosome 13q deletions in regions that contain the *NALCN* gene (Kirchhoff et al., 2009; Lalani et al., 2013). Recently, a whole genome linkage analysis in three-generations of a south Indian family who had multiple members affected with juvenile myoclonic epilepsy (JME) found a critical genetic interval of 24 Mb between markers D2S116 and D2S2390 (Ratnapriya et al., 2010). The *UNC-80* gene lies in this region. In addition, a region-wide linkage analysis carried out in 118 European families with an aggregation of JME indicated a significant linkage with marker rsD2S143 located on chromosome 2q34 at around 4Mb of the *UNC-80* gene (EPICURE Consortium et al., 2012).

### Autism

Autism Spectrum Disorders (ASDs) are neurodevelopmental disorders characterized by impairments in social interaction and communication, and the presence of restrictive and repetitive behaviors (reviewed in Jones et al., 2014). Exome sequencing of 343 families, each with a single child on the autism spectrum and at least one unaffected sibling, revealed *de novo* small indels and point substitutions in the affected sibling (Iossifov et al., 2012). One of the discovered mutations resulted in a truncating R518X mutation in one copy of *UNC-80*, suggesting that *UNC-80* is a susceptibility locus in autistic spectrum.

### 13q syndrome

The 13q syndrome is caused by structural and functional monosomy of the 13q chromosomal region and was first described in 1963 and consequently delineated as a specific syndrome in 1969 (Lele et al., 1963; Allderdice et al., 1969). Large numbers of patients with deletions of the long arm of chromosome 13, which includes *NALCN* and *NLF-1*, have been described in the literature. The phenotypes of these patients varied widely. Some showed severe malformations involving the brain, heart, kidneys, lungs, other organ systems, and digits while others were only mildly affected with minor dysmorphic features, developmental delay, and growth failure. The characteristic features of 13q deletions include growth retardation with microcephaly, facial dysmorphism, congenital heart, brain, and kidney defects (Brown et al., 1995). Several studies addressed karyotype-phenotype correlations depending on the extent of the deletions (Brown et al., 1995; Luo et al., 2000; McCormack et al., 2002; Alanay et al., 2005; Garcia et al., 2006; Ballarati et al., 2007; Walczak-Sztulpa et al., 2008; Kirchhoff et al., 2009; Quelin et al., 2009). It was reported that the severely malformed phenotype results from the deletion of a critical region lying between markers D13S136 and D13S147 (Brown et al., 1995). This region of around 2.3 Mb contains several genes including *NALCN*. Recently, a molecular-karyotyping study reported that a female with congenital heart defects, facial anomalies, developmental delay, and intellectual disability had a deletion of 12.75 Mb extending from 13q33.1 to 13q34 (Huang et al., 2012). The deleted region harbors 55 genes, including *NALCN* and *NLF-1*. Another study further refined a critical region for all CNS anomalies and neural tube defects taken together, to a region of 1.6 Mb (Kirchhoff et al., 2009). Only seven known genes are located within this region, including *NALCN*. A recent study aiming to identify rare DNA copy number variants in cardiovascular malformations with extracardiac abnormalities revealed the deletion of 641 kb in the *NALCN* gene region in 3 patients (Lalani et al., 2013). The extracardiac phenotypes of these patients include agenesis of corpus callosum, growth retardation, facial dysmorphism, and seizures.

### Alzheimer's disease

Alzheimer's disease (AD) is the most common form of dementia and the most frequent degenerative brain disorder encountered in old age. The risk of developing AD substantially increases after 65 years of age (reviewed in Nussbaum and Ellis, 2003). With the exception of rare cases of early onset familial Alzheimer's disease caused by mutations in the amyloid precursor protein or presenilins genes, the etiology of the vast majority of cases remains misunderstood. There is evidence for substantial genetic influence (reviewed in St. George-Hyslop and Petit, 2005; Tanzi and Bertram, 2005). A linkage analysis to detect novel AD loci from 437 families (1252 individuals) revealed a statistically significant linkage with marker D2S2944 located in 2q34 in 31 American families with a minimum age at onset between 50 and 60 years (Scott et al., 2003). This marker is located at less than 4Mb from the *UNC-80* gene. Another study reported the existence of a susceptibility locus in 14q32.12 near marker D14S617 in a Caribbean Hispanic cohort of 1161 individuals from 209 families (Lee et al., 2008). The *UNC-79* gene lies in a region at less than 3Mb from this marker. The existence of a susceptibility locus for AD in the vicinity of the *UNC-79* gene (SNP rs11622883) was also reported by Grupe et al., 2007, from a cohort of 1808 American-European patients (Grupe et al., 2007).

### Alcoholism

Alcohol dependence is one of the most common and costly public health problems. Several studies suggest a role of genetic factors and few genes have been shown to be associated with alcohol dependence (reviewed in Morozova et al., 2014). A recent GWAS performed in 118 European-American families (2322 individuals) demonstrated a significant linkage of SNP rs17484734, located in the *NALCN* gene, and high-risk of alcohol dependence (Wetherill et al., 2014). Interestingly, mice that heterologously carry a hypomorphic mutation in the *Unc-79* gene voluntarily consume more ethanol than wild-type littermates (Speca et al., 2010). A GWAS on comorbid alcohol/nicotine dependence in 599 cases and 488 controls found SNP rs12882384 as one of the three top findings, which is located within the *UNC-79* gene (Lind et al., 2010). Genome-wide significance was found for joint alcohol/nicotine comorbidity but not for nicotine or alcohol dependence alone suggesting the existence of genes that mediate the combined effect of these two addictive substances. In addition, two previous studies suggest the existence of a susceptibility locus located on chromosome 2, near the *UNC-80* gene, linked to alcohol tolerance (markers D2S425, D2S434, D2S424, D2S1323, D2S1333) and the comorbidy of alcoholism and depression (marker DS1371) respectively (Nurnberger et al., 2001; Schuckit et al., 2001).

### Restless legs syndrome

Restless legs syndrome (RLS) is a sensorimotor disorder characterized by abnormal sensations in the limbs that are both dependent on activity and time of day, such that symptoms are promoted by rest and relieved by activity and peak in the evening or at night (reviewed in Winkelman et al., 2013). RLS is a genetically heterogeneous complex trait with high prevalence but large phenotype variability. Current theories of RLS pathophysiology emphasize brain iron deficiency with abnormal dopaminergic consequences, together with a strong underlying genetic background (reviewed in Dauvilliers and Winkelmann, 2013). A recent study analyzing 11 individuals from the same family indicated the existence of a locus on chromosome 13q, between SNPs rs2182885 and rs7333498 (around 5.3 Mb in size; Balaban et al., 2012). The *NALCN* gene lies in this region.

### Primary biliary cirrhosis

Primary biliary cirrhosis is a chronic granulomatous cholangitis, characteristically associated with antimitochondrial antibodies. Analysis of both aggregation data from families and concordance data from twins revealed a genetic predisposition for the disease (reviewed in Hirschfield and Gershwin, 2013). A GWAS with a cohort of 2072 North-American individuals (536 patients and 1536 controls) was carried out by genotyping more than 300,000 SNPs (Hirschfield et al., 2009). Although this study reported the strongest association of the disease with HLA-related genes, a significant association was found with SNP rs2211312 in the *NLF-1* gene.

### Hypertension and blood pressure

Hypertension is a common human disease affecting over one billion people world-wide and a major contributor to cerebrovascular accidents, myocardial infarction, congestive cardiac failure and chronic renal failure (reviewed in Zhao et al., 2013). A GWAS performed by genotyping more than 800,000 SNPs in a sample of 1017 African-American individuals led to the identification of several potential loci involved in the regulation of blood pressure (Adeyemo et al., 2009). A significant association was found between systolic blood pressure and SNP rs9301196 located within the *NLF-1* gene.

### Polyglutamine disorders

Polyglutamine (polyQ)-expansions in different proteins cause at least eleven neurodegenerative diseases including Huntington disease and spinocerebellar ataxias (reviewed in Todd and Lim, 2013). These human diseases are caused by extreme expansion of the repeats, which adversely impact protein structure, often causing intracellular protein aggregation and altered protein function. Bovine NLF-1 exhibits a polymorphic polyQ tract in its sequence (Whan et al., 2010). This polyQ tract is also found in other species including human beings suggesting that aggregation of NLF-1 could be involved in neurodegenerative diseases.

### Cancer

Biankin et al. examined pancreatic cancer genomes using exome sequencing and copy number analysis from a cohort of 142 patients of early (stages I and II) sporadic pancreatic ductal adenocarcinoma (Biankin et al., 2012). Analysis of genes with non-silent mutations occurring in 2 or more individual cancers identified 16 genes including *NALCN* (mutated in 4 patients). Pathway analysis allowed not only the identification of genes involved in mechanisms already known to be involved in cancer but also genes known to be involved in axon guidance. This latter finding was strengthened by the inclusion of data from another study (Jones et al., 2008). It is tempting to speculate that *NALCN* could also be involved in axon guidance.

An association of *NALCN* with non-small cell lung cancer was also suggested by a study where 217,817 SNPs were genotyped in 348 advanced patients who received chemotherapy (Lee et al., 2013). These genetic association studies revealed that SNP rs9557635, located in the genomic regions of the *NALCN* gene, was associated with this disease. Interestingly, this study also reported a strongly significant association with SNP rs2371030 located 25 kb downstream to the *CPS1* gene on chromosome 2q34, which contains the *UNC-80* gene. Further linking the channelosome to cancer, a recent study reported an *NLF-1* copy number increase in populations of tumor-derived endothelial cells that are resistant to anti-angiogenic cancer therapies (McGuire et al., 2012). Finally, in a search for key altered genomic regions in human glioblastomas, Fontanillo et al., 2012, found a high correlation between the malignant state and copy number alterations in the *NALCN* and *NLF-1* regions with a decrease in expression level (Fontanillo et al., 2012).

## Concluding remarks and future directions

### Is NALCN really a channel *per se*?

From a fundamental point of view, it remains unclear whether NALCN is truly an ion channel. The early findings by Ren and colleagues supported that NALCN was an ion channel, based on mutations in the putative selectivity filter (EEKE) that alter the permeation properties and the gadolinium block (EEKA) or inhibit the observed currents in HEK-293 cells (EEEE)(Lu et al., 2007). Further supporting this, in *D. melanogaster*, the expression of a *na^EEEE^* mutant in *na* neurons using the UAS-GAL4 system did not restore the studied phenotype as *na^wt^* does (Lear et al., 2005). However, functional expression of NALCN channel in heterologous expression systems is difficult to obtain and these data do not demonstrate unambiguously that NALCN is a pore forming subunit. A clear proof of its channel activity would come from electrophysiological data exploring NALCN channel activity when rebuilt in lipid bilayers.

### What are the gating properties of NALCN?

The mechanism(s) that gate NALCN have yet to be established. On one hand, NALCN channel is described as conducting a sodium leak current in neurons both in mammals and invertebrates (Lu et al., 2007; Lu and Feng, 2011; Xie et al., 2013). On the other hand, NALCN channel also conducts a sodium current activated by acetylcholine though the M3R in a pancreatic β-cell line without any evidence for a leak current (Swayne et al., 2009). One may hypothesize that the NALCN-mediated leak current observed in neurons may result from a constitutive activity of some GPCRs (Swayne et al., 2009). In this case, it would require UNC-80 and SFKs. However, this hypothesis may not be valid for two main reasons. First, considering that HEK-293 cells do not express *UNC-79* and *UNC-80* (Swayne et al., 2009), the expression of NALCN alone is sufficient to observe a sodium leak current in these cells (Lu et al., 2007). Second, it was shown that a sodium leak current mediated by NALCN is still present in hippocampal neurons from *Unc-79* knockout mice where UNC-80 is not detected (Lu et al., 2010). Thus, the gating properties of NALCN remain mysterious and should be investigated further. In keeping with this idea, the search for additional proteins that belong to the NALCN channelosome could give clues regarding modulation of the NALCN gating mechanism.

### What is the repertoire of GPCRs capable of modulating NALCN channel?

Recent studies have established that NALCN can be activated/potentiated or inhibited by different GPCRs (Lu et al., 2009; Swayne et al., 2009; Lu et al., 2010). Therefore, one may expect additional GPCRs to be involved in these processes. Identification of this repertoire is an important challenge and understanding why some GPCRs modulate NALCN, and some others do not, would help to understand further how NALCN is activated/regulated in neurons and how neurotransmitters and hormones affect neuronal electrophysiological properties.

### What are the functional consequences of alternative splicing on NALCN channelosome genes?

Alternative splicing events were demonstrated in many ion channels and can significantly impact their biophysical properties, cellular localization, and functional regulations (reviewed in Noel et al., 2011; Jan and Jan, 2012; Lipscombe et al., 2013). Alternative splicing was identified in NALCN channelosome genes (Senatore et al., 2013; Monteil *unpublished data*). The physiological consequences of these alternative splicing events remain to be elucidated.

### Are UNC-79, UNC-80, and NLF-1 specific NALCN-ancillary subunits?

In *C. elegans, nlf-1, unc-79, unc-80, and nca-1;nca-2* mutants exhibit exactly similar phenotypes and both *Unc-79* and *Nalcn* knockout mice die soon after birth. However, the post-natal lethality observed in these mouse models could mask phenotypes more specific to one subunit compared to the others. As a matter of fact, we cannot exclude that UNC-79, UNC-80, and NLF-1 may have NALCN-independent physiological roles. Conditional knockout mice for each component of the NALCN channelosome would be very helpful to clarify this point.

### Are there more proteins in the NALCN channelosome?

The NALCN channelosome is probably not restricted to NALCN, UNC-79, UNC-80, NLF-1, SFKs, and GPCRs. As an example, there is another gene highly related to *NLF-1, FAM155B* in mammals (accession number NG_021282.1). Whether the *FAM155B* gene product belongs to the NALCN channel complex should be investigated. Also, the mechanisms involved in the regulation of the NALCN channel by CaSR are not known and could imply a co-inclusion in the same protein complex. Some studies indicate genetic interactions between *nca-1;nca-2* and other genes in *C. elegans*. As a matter of fact, null mutants of stomatins (*unc-1* and *unc-24*) and innexins (*unc-7* and *unc-9*) were found to restore the altered sensitivity to GAs and ethanol (Sedensky and Meneely, 1987; Morgan et al., 1990; Morgan and Sedensky, 1995; Sedensky et al., 2001; Humphrey et al., 2007). It was also found that *nca-1;nca-2* mutants exhibit a reduced evoked post-synaptic current at the neuromuscular junction and this phenotype is rescued by *unc-7* mutants (Bouhours et al., 2011). However, in this latter study, co-immunofluorescent staining experiments do not show obvious neuronal co-localization of UNC-7 and NCA-1/NCA-2. Thus, the reported genetic interaction between *unc-7* and *nca-1;nca-2* probably reflects a functional, rather than a physical interaction. A genetic interaction was also described between *unc-26* null mutants and *unc-80* and *nca-1;nca-2* (Jospin et al., 2007). *unc-26* encodes for Synaptojanin, a lipid phosphatase required to degrade phosphatidylinositol 4,5 bisphosphate (PIP2) at cell membranes during synaptic vesicle recycling (Cremona et al., 1999). It was found that *unc-80* and *nca-1;nca-2* mutants ameliorate the recycling defects of synaptic vesicles as well as the synaptic transmission in *unc-26* null mutants and it was suggested that *nca-1;nca-2* function could be regulated by PIP2 (Jospin et al., 2007). Whether Synaptojanin belongs to the NALCN channelosome remains to be investigated. Interestingly, *NALCN* and *PLA2G6* mutations results in similar phenotypes in humans suggesting that iPLA2β could be a regulator of NALCN. Complementary techniques such as two-hybrid and immunoprecipitation studies followed by mass spectrometry approaches will certainly lead to the discovery of new components of the NALCN channelosome.

### How is NALCN functions modulated?

In addition to their regulation by binding partners, ion channels are known to be post-translationally modulated. For example, ion channel complexes can be phosphorylated by a variety of kinases which regulate their physiological roles (reviewed in Smart, 1997; Dai et al., 2009; Cerda et al., 2011; Scheuer, 2011; Vacher and Trimmer, 2011). An *in silico* analysis of primary amino-acid sequences of NALCN, UNC-79, UNC-80, and NLF-1 reveals the existence of several predicted phosphorylation sites that are conserved in mammals and for some of these sites, are also conserved in *C. elegans, D. Melanogaster*, and *L. Stagnalis* (*not shown*). The physiological relevance of the NALCN channelosome phosphorylation, as well as the identification of the kinases involved, remains to be explored.

### In which neuronal subtype(s) is NALCN expressed?

*NALCN*, and probably *UNC-79, UNC-80*, and *NLF-1*, is widely expressed throughout the CNS in mammals (Lee et al., 1999; Swayne et al., 2009; Kang et al., 2011). To better assess the physiological role of NALCN, it is crucial to identify whether expression is restricted to some specific neuronal populations. In addition, NALCN is localized along the axons in *C. elegans* and in synaptic regions in *D. melanogaster* (Nash et al., 2002; Humphrey et al., 2007; Yeh et al., 2008), but the neuronal localization of NALCN channel proteins in mammals is unknown. The development of specific antibodies for immunohistochemistry experiments will be crucial for this purpose.

### Lack of pharmacology of NALCN but relevance of NALCN as a drug target

To date, no specific pharmacology for NALCN has been reported and this clearly represents a significant hurdle in the study of NALCN properties and activity. The discovery of specific NALCN agonists/antagonists is critically needed. Such molecules would be tools to investigate NALCN's physiological roles and may also hold interest to develop innovative therapeutic strategies. Whether the NALCN channelosome represents a drug target of interest remains unclear at the present time. Considering that NALCN channel is expressed in pancreatic islets, both in α- and β-cells, and is possibly involved in the regulation of insulin secretion (Kutlu et al., 2009 and http://t1dbase.org; Swayne et al., 2010), targeting NALCN might be relevant to treat type 2 diabetes. It is tempting to speculate that agonists of NALCN would have beneficial effects in pancreatic β-cells on insulin secretion (reviewed in Gilon and Rorsman, 2009). NALCN blockers might be relevant to treat pain considering that SP plays a pivotal role in the transmission of noxious stimuli in the spinal cord (reviewed in Seybold, 2009) and that *NALCN* is highly expressed in the dorsal spinal cord compared to the ventral spinal cord (Sun et al., 2002). Based on the potential implication of NALCN channel in human physiology and diseases, its pharmacological targeting might also be relevant to treat epilepsy, bowell motility disorders, osmoregulation disorders and psychiatric disorders.

### Summary

The NALCN channelosome is vital in mammals: homozygous mutant mice exhibit neonatal lethal phenotype and NALCN plays a crucial role in neuronal excitability. NALCN is involved in the regulation of resting membrane potential and is modulated by hormones and neurotransmitters. In the present article, we provide an extensive review of the literature describing the molecular and functional properties of the NALCN channelosome, the various phenotypes observed in NALCN animal models, as well as the recent implications of NALCN in human diseases. We also discuss how NALCN could represent a therapeutic target to treat a wide variety of human diseases, especially neurological and psychiatric diseases. Unfortunately, the lack of specific pharmacology is clearly a brake to all the NALCN investigations. In the coming years, the expected development of selective agonists/antagonists, as well as new animal models, should foster our understanding of the functional properties and physiological roles of the NALCN channelosome.

### Conflict of interest statement

The authors declare that the research was conducted in the absence of any commercial or financial relationships that could be construed as a potential conflict of interest.
